# Pain Treatment in the Companion Canine Model to Validate Rodent Results and Incentivize the Transition to Human Clinical Trials

**DOI:** 10.3389/fphar.2021.705743

**Published:** 2021-08-05

**Authors:** Michael J. Iadarola, Dorothy Cimino Brown, Alexis Nahama, Matthew R. Sapio, Andrew J. Mannes

**Affiliations:** ^1^ Department of Perioperative Medicine, Clinical Center, NIH, Bethesda, MD, United States; ^2^ Elanco Animal Health, Greenfield, IN, United States; ^3^ Ark Animal Health, San Diego, CA, United States

**Keywords:** cancer pain, osteoarthritis pain, resiniferatoxin (RTX), TRPV1, transient receptor potential vanilloid type 1 receptor, opioid, palliative analgesia, activity monitoring

## Abstract

One of the biggest challenges for analgesic drug development is how to decide if a potential analgesic candidate will work in humans. What preclinical data are the most convincing, incentivizing and most predictive of success? Such a predicament is not unique to analgesics, and the pain field has certain advantages over drug development efforts in areas like neuropsychiatry where the etiological origins are either unknown or difficult to ascertain. For pain, the origin of the problem frequently is known, and the causative peripheral tissue insult might be observable. The main conundrum centers around evaluation of translational cell- and rodent-based results. While cell and rodent models are undeniably important first steps for screening, probing mechanism of action, and understanding factors of adsorption, distribution metabolism and excretion, two questions arise from such studies. First, are they reliable indicators of analgesic performance of a candidate drug in human acute and chronic pain? Second, what additional model systems might be capable of increasing translational confidence? We address this second question by assessing, primarily, the companion canine model, which can provide particularly strong predictive information for candidate analgesic agents in humans. This statement is mainly derived from our studies with resiniferatoxin (RTX) a potent TRPV1 agonist but also from protein therapeutics using a conjugate of Substance P and saporin. Our experience, to date, is that rodent models might be very well suited for acute pain translation, but companion canine models, and other large animal studies, can augment initial discovery research using rodent models for neuropathic or chronic pain. The larger animal models also provide strong translational predictive capacity for analgesic performance in humans, better predict dosing parameters for human trials and provide insight into behavior changes (bladder, bowel, mood, etc.) that are not readily assessed in laboratory animals. They are, however, not without problems that can be encountered with any experimental drug treatment or clinical trial. It also is important to recognize that pain treatment is a major veterinary concern and is an intrinsically worthwhile endeavor for animals as well as humans.

## Introduction

### Background and Scope

The companion canine model in pain research is a relatively new addition to the field of analgesia research. In this report we examine the foundational studies that led us to develop the companion canine model, the evolution of this model, provide examples of its use in translation of novel analgesics to phase one trials, outline various other aspect of the model in terms of the pain research ecosystem, and briefly discuss other aspects and other large animal models that may provide potential insights into new indications for analgesic use.

Development of the companion canine model Karai et al. (2004), Brown et al. (2005) began subsequent to our discovery of the calcium overload mechanism for selective inactivation of TRPV1-expressing neurons, axons, or nerve terminals by RTX (Olah et al., 2001). TRPV1 is a heat and inflammation sensitive ion channel Caterina et al. (1997) that is highly expressed by nociceptive primary afferents Cavanaugh et al. (2011a), Cavanaugh et al. (2011b), particularly Aδ and C-fiber nociceptive neurons in the dorsal root and trigeminal ganglion (Mitchell et al., 2014). The companion dog model was developed to test this novel mechanism in a species and a pain condition more closely aligned clinical pain problems in humans, rather than transition from analgesic rodent results directly to human trials. After evaluation of RTX, use of the companion dog model was extended to analgesic assessments of a protein therapeutic agent Substance P-Saporin (Mantyh et al., 1997; Allen et al., 2006; Brown and Agnello, 2013). Saporin is a plant-derived toxin that inhibits protein synthesis. Attachment of the neuropeptide Substance P directs the conjugate to substance P receptor-expressing neurons. When injected into the CSF around the spinal cord the toxin-peptide conjugate is internalize by agonist-mediated receptor endocytosis and the toxin is eventually delivered to the cytoplasm where it stops protein synthesis (Yaksh et al., 2017). This mechanism is used to delete second order spinal cord neurons that are critical in transmitting nociceptive signals to the brain. Again, a novel approach to pain control that could benefit from evaluation in a veterinary canine clinical pain model. The ultimate translation to humans of these two agents led to two divergent results: for the small molecule ion channel activator translation has progressed to multiple clinical trials (NCT00804154; NCT02522611; NCT03542838) whereas the protein therapeutic underwent an abbreviated trial in human cancer pain patients but ended without positive findings (NCT02036281). The crux of the matter partially resides in the *details of drug administration* and tissue distribution and localization of the target receptor. Both agents are interventional and require precise injections at, or near, the site of the intended target. The elements of successful administration and mechanisms of action will be examined in detail.

This review mainly focuses on these two interventional agents and their use in canine disorders that exhibit a prominent pain component. We will not examine to any great extent studies performed on cats, and the reader is referred to other reviews to access the feline literature (Klinck et al., 2017; Birder and Andersson, 2018; Klinck et al., 2018; Lascelles et al., 2018). However, we briefly consider work done in swine, goats and horses. We also acknowledge that, in the course of drug development efforts, many studies of analgesic agents are conducted with purpose-bred dogs, especially to fulfill the toxicology requirements of an Investigational New Drug (IND) application to the Food and Drug Administration (FDA). However, the results of such evaluations, while undeniably important, frequently do not include investigation of efficacy in a companion canine pain disorder, are generally proprietary, and are often unpublished. While some published toxicological studies are referred to throughout this review, improved access to such data may facilitate future analgesic drug development efforts.

**FIGURE 1:**
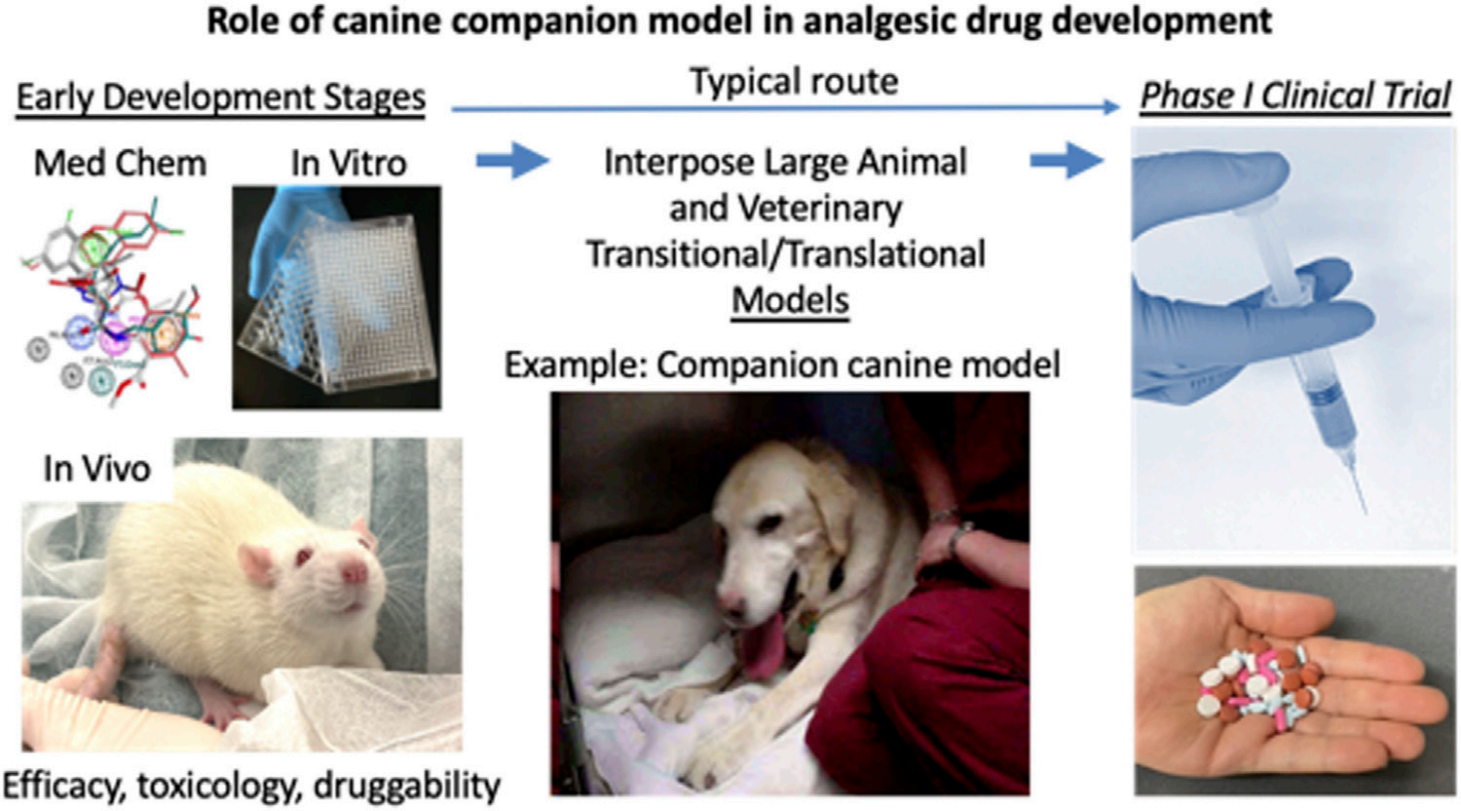
Role of companion canine model in analgesic drug development. The drug development process progresses from left to right. The early development steps include, discovery, medicinal chemistry, *in vitro* screening, *in vivo* testing in rodent models, and optimization for target engagement, adsorption, distribution, metabolism, excretion. Usually, after manufacturing processes and toxicology are conducted, the candidate molecule goes into phase I testing. What is often lacking, despite positive result from mouse or rat studies is a clear indication of whether the compound produces analgesia in clinical pain states. These would be indications arising from natural diseases. To address this gap the veterinary companion canine model is interposed between the two major milestones in drug development. The two main clinical conditions that have been used for analgesic drug evaluation are pain from osteosarcoma and osteoarthritis. These conditions both impact motor activity and performance which provide obvious endpoints for evaluation. Since the pain from the conditions affects activities of daily living, the owners have multiple parameters from which to judge improvement in their animal’s behavior. Formal, validated scales have been developed for evaluation of both pain severity and pain interference with activity (Brown et al., 2007; Brown et al., 2008; Brown et al., 2009; Brown et al., 2010; Brown et al., 2013a; Brown et al., 2013b; Brown, 2014). Other parameters available include objective measures such as force plate determinations of weigh bearing and analysis of video recordings. Because a weight bearing limb (or limbs) is involved in both disorders and either a fore or hind limb can be affected, gait and force plate measurements sometimes can be difficult to obtain in a reproducible or reliable manner (Iadarola et al., 2018). However, the range of potential available endpoints is broad enough to be adapted to a variety of conditions.

*Where Do Companion Models Fit in Drug Development Schemes?* One of the main questions translational pain scientists might have about the topic of large animal models is why and in what ways are canine models superior to current approaches? The answer is that these models are not necessarily superior, rather they play a different role in the development process than rodent models. One place for the companion canine model in translational drug development efforts is shown in Figure 1. When interposed between the early developmental stages and a potential phase one clinical trial, positive canine results provide strong validation of preceding cellular and rodent model data. This is especially true for the interventional toxin approaches outlined here. As noted, the idea of stepping from rodent chemo-axotomy or neuronal deletion an equivalent trial in human spinal cord or ganglion is a large leap despite obtaining strong efficacy and safety in rodents and healthy laboratory canines. The use of companion canine models does not need to be a sequential step in the development process. Many questions can be answered to improve human phase two and phase three clinical trial designs by using companion animals in parallel with the conduct of an ongoing phase one or two trial. Examples include evaluations of drug/drug interactions, dosage protocols (frequency, escalation paradigms), and side effect monitoring; all can inform and accelerate human drug development if deliberately thought out and implemented early on. Our experience the more novel interventional agents was that evaluating performance of these approaches in a transitional model closer to the human greatly facilitated our understanding of administration procedures, dosing parameters and safety well in advance of both the IND campaign and first-in-man human clinical trials in the intrathecal compartment. The companion canine model filled this critical gap by treating naturally occurring disease in (often elderly) animals with pre-existing conditions. Beyond this, the anatomy of a dog or pig is much more comparable to human, and better suited to examining interventional agents. There are also therapeutic gaps for effective treatments for animals with painful conditions including palliative care for cancer and control of osteoarthritis pain that need to be filled. Other than opioids or NSAIDS, no other effective treatment for pain from advanced cancer is available, and less than satisfactory pain control is available for arthritis. This situation is identical to that in humans and the use of companion canine models allows the unmet needs of *both* species to be addressed. Some of the advantages of the companion canine model and comparison to rodent models are listed in Table 1.

**TABLE 1:** Advantages of companion animal models.

Canine model	Attributes and elements	Rodent models
Owner is familiar with animal’s activities of daily living (ADL)	Activity levels eating activities and food preferences sleeping, gait and movement	Monitoring of activity is possible in the home cage, open field or by other devices
Owner is familiar with animal’s “personality”	Emergence of neurological or “psychiatric” problems may be evident	Must actively assess presence of anxiety or depression
Naturally occurring diseases (actually not models)	Pathologically similar to human disorders and occur over a long time period. No need for artificial inducing agents	Needs inducing agents, e.g., carrageenan inflammation or iodoacetate or kaolin for joint osteoarthritis models
Exhibit complex behaviors spontaneously	Pets will seek out the owner and respond to family or visitors, validated outcome measures are available	Exhibit complex behaviors, need experimental settings or apparatus for measurement
Many breeds, genetic diversity	Higher prevalence of disorders in some breeds can be used to enrich for recruitment	Outbred strains or inbred strains can be used to explore specific traits
Tissues more similar to human	At the histological level, dog cells are more similar to human than are rat cells	Tissues less similar to human. There are differences in transcriptome between the 3 species iadarola et al., (2018)
Efficacy measures can be judged by multiple observers in the home or clinic	Nearly continuous monitoring is possible. Can be correlated with activity monitor readouts	Efficacy generally measured by evoked responses to acute stimuli, inflammation and incision models for hyperalgesia, models of joint pain, chemotherapy induced peripheral neuropathy etc. Brown et al., (2008), Brown et al., (2013a), Brown et al., (2013b)
Allows better estimation of starting dose in human phase I trial	The doses used in the companion animal model were almost identical to those use in human trials for cancer pain and osteoarthritis	Provide initial dose-response of candidate drugs that may approximate starting dose in humans
Positive results provide strong incentive to proceed to human clinical trials or provide an indication for a “no go” signal	This is an important advantage. Clear-cut analgesia in the canine companion model is a strong predictor of positive translation in human trials	Positive results begin the process of translation

## RESINIFERATOXIN FOR INTERVENTIONAL, PERSONALIZED PAIN MANAGEMENT 

### Foundational Studies Defining the RTX Mechanism of Action

The eventual clinical use of RTX had its beginning in our basic research into the question: How does pain start? It was well appreciated that nerve endings and axons in the skin and deep tissues are the physical, cellular-level structures that receive and transmit painful stimuli originating from frank tissue damage or external sources (e.g., heat). However, the range of effector molecules in the nerve endings responsible for transducing noxious stimuli into electrical impulses remained to be defined at the molecular level. In 1997, the TRPV1 receptor was cloned by Caterina et al. (1997), and starting from this sequence, a full-length cDNA was isolated from rat dorsal root ganglion and we made a fusion protein with the mammalian codon-optimized “enhanced” Green Fluorescent Protein (TRPV1-eGFP) (Olah et al., 2001). We used the fusion protein to make a stably expressing cell line for live cell imaging. At that time, our experiments were more curiosity-driven rather than consciously translational. TRPV1 conducts calcium ions and, as an aside, making the cell line required use of a weak promoter to prevent overexpression of TRPV1. We found that overexpression with the cytomegalovirus (CMV) promoter caused the cells to become leaky to calcium and undergo apoptosis. Thus, in the first set of cultures using the CMV plasmid, cells that retained the TRPV1 insert were negatively selected and those that recombined the cassette to remove the TRPV1 portion of the insert but retain the antibiotic resistance gene were positively selected. Substitution of a minimal metallothionein promoter for the much stronger CMV promoter generated a viable cell line that expressed visible amounts of the TRPV1-eGFP fusion protein that was functional and produced a robust calcium influx upon activation (Olah et al., 2001).

Based on observations made by vital imaging of the stable TRPV1-eGFP cell line and primary cultures of DRG neurons (many of which express TRPV1) it became apparent that binding of RTX to TRPV1 opened the ion channel and induced a substantial calcium overload. Exposure of the culture to RTX could kill the cells in a matter of minutes Olah et al. (2001) and RTX was much more potent compared to capsaicin, ∼500 times in this assay (Kárai et al., 2004). The idea of using RTX to effectively manipulate, in fact, literally kill TRPV1 neurons or their processes for pain control became apparent from watching RTX-induced calcium cytotoxicity on the stage of a microscope using live cell imaging of the TPRV1-eGFP cell line (Olah et al., 2001). This result launched a series of *in vivo* studies in rats in which the effects of RTX on peripheral nerve endings in hind paw Pecze et al. (2009), Cruz et al. (2014), Raithel et al. (2017), TRPV1 neurons in trigeminal and lumbar dorsal root ganglia Karai et al. (2004), and perineural routes of administration Neubert et al. (2008) were explored. In all of these cases RTX produced significant and substantial analgesic activity Iadarola and Mannes (2011), Iadarola and Gonnella (2013) and these results formed the underlying impetus for development of the companion canine model and for a human clinical trial in cancer pain.

### Preclinical Animal Models for Translation of RTX

Once the mode of RTX action was clarified and preclinical rodent studies showed efficacy, the idea of conducting a human clinical trial by injecting RTX somewhere along the route of the nociceptive primary afferent neuron gained momentum. As noted, there were reservations about killing cells in the DRG centering around the possibility of generating a denervation hyperesthesia or a synaptic rearrangement in the spinal cord dorsal horn that would be deleterious in some way. We had not seen this in the rat studies. Even when we unilaterally injected RTX directly into the trigeminal ganglion Karai et al. (2004) we did not observe abnormal scratching behavior directed towards the facial dermatomes nor did we see a unilateral neglect syndrome. The analgesia also was durable and did not diminish over time. Using the capsaicin eye wipe test, no loss of analgesic efficacy was detected in a group of trigeminally injected rats observed for a full year Karai et al. (2004) and no impairment of the blink reflex was seen Karai et al. (2004), Tender et al. (2005), Bates et al. (2010), indicating fiber-type selectivity of RTX treatment. Long duration results also were obtained with lumbar intrathecal injections and inhibition of hind paw thermal nociception as the endpoint. In the paw, thermal analgesia occurred without affecting mechanical withdrawal in the von Frey hair test. These rodent studies formed the basis for additional transitional studies in monkey and dog.

RTX injections made directly into the trigeminal ganglion of rhesus macaques further impelled the therapeutic development of RTX (Tender et al., 2005). Here, target engagement in the trigeminal system was assessed by the capsaicin eye wipe test. As in the rat, unilateral trigeminal injection produced a unilateral loss of capsaicin-induced eye wipe. The analgesic effect was durable and lasted at least 3 months. There was also a loss of peripheral plasma extravasation indicating that TRPV1 neuronal cell bodies had been exposed to RTX and were deleted by the intraganglionic injection. This was confirmed upon necropsy and immunocytochemical staining of the ganglion. A significant loss of TRPV1-immunoreactive neuronal perikarya was observed. The analgesia and TRPV1 neuronal deletion occurred without affecting healing of the scalp incision, other facial functions, or swallowing or feeding. Again, these results, while strongly encouraging, were still in the arena of experimental nociception rather than a test in a spontaneously occurring disease entity.

**FIGURE 2:**
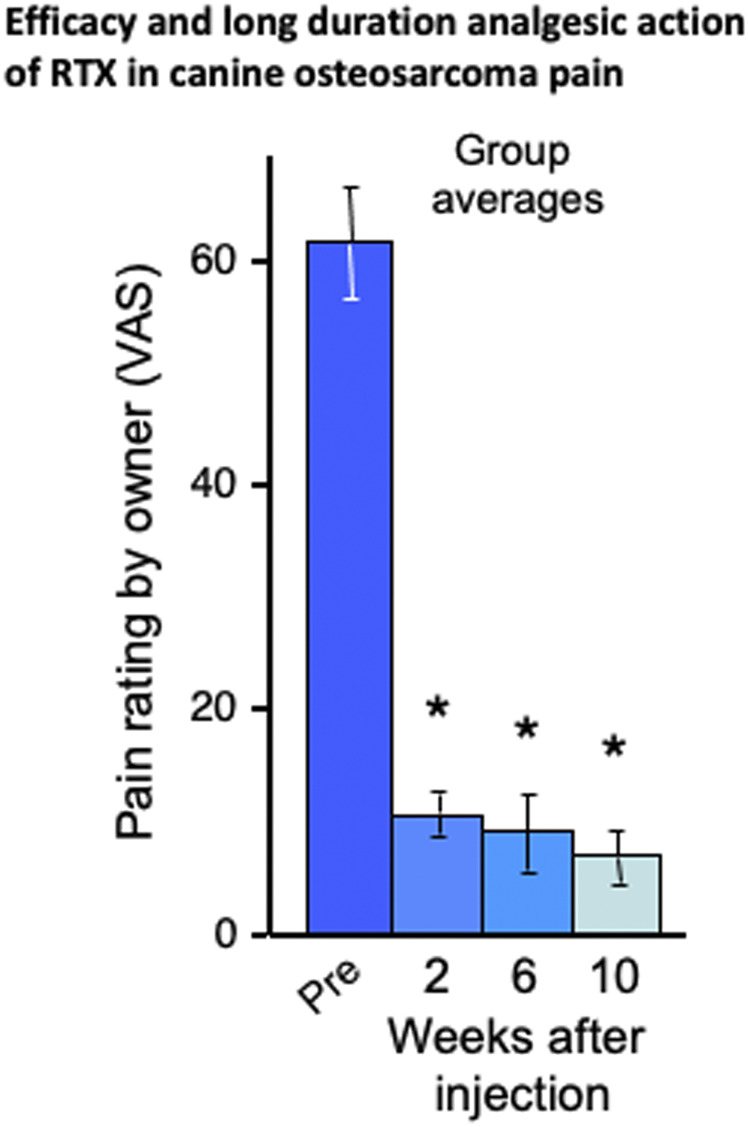
Efficacy and long duration analgesic action of RTX in companion canine model. RTX analgesia was tested in naturally occurring neoplasms and/or osteoarthritis in the dog after a single intrathecal dose of a single intrathecal dose of RTX (1 μg/kg) administered under general anesthesia via cisternal puncture. Reports by the owners using Visual Analog Scale rating demonstrated a sustained reduction in pain in the eight dogs. The bars represent the average rating ±SEM (*ANOVA with Scheffe’s post hoc test; *p* < 0.05). The animals initially presented with limb guarding as they walked, but this improved over time and daily activity increased, as was evident from video recordings. In fact, the entire demeanor of the dogs appeared improved following intrathecal RTX treatment. The efficacy of RTX action was further demonstrated by discontinuation or reduction in the use of supplementary analgesics (opioids and NSAIDs in all eight dogs). As the time points on the *X* axis show, the RTX-induced analgesia did not diminish despite neoplastic advancement. These data formed a bridge between the initial rodent data Karai et al. (2004) and the human clinical trials in cancer pain as depicted schematically in Figure 1 and with preliminary human result in Figure 3.

This knowledge gap was rectified in the first canine study which tested the efficacy of intrathecal RTX in eight companion dogs with osteosarcoma, debilitating osteoarthritis, or both (Figure 2). The owners had brought their animals to the veterinary clinic because pain was not well controlled after exhausting standard of care options. For these animals a single 1 μg/kg dose of RTX was infused over 10 min into the cisterna magna. Pain intensity was rated by the owners on a 0 to 10 visual analog scale before, and at 2, 6, and 10-weeks post injection. A significant analgesic action was observed at each post-injection time point and amounted to an ∼85% decrease compared to the pre-injection pain rating (Figure 2 , from Karai et al., 2004). These data show that the analgesic action of a single injection was durable and lasted for the remainder of the animal’s lifetime. In that same study, the actions of RTX were also examined at the cellular level *in vitro* in primary cultures of human DRG neurons. RTX produced a marked and sustained increase in calcium influx in a subpopulation of DRG neurons but nearby, nonresponding, non-TRPV1-expressing neurons maintained normal calcium levels. This further added to the idea that RTX could be used as an interventional analgesic in humans. These data represented a progressive series of steps from cell-based assays, to rodent models, to clinical pain in companion canines, to human DRG neurons but a key element in the decision process to proceed to human clinical trials (NCT00804154) were our observations of reproducible and sustained analgesic efficacy, without development of behaviors indicative of denervation hyperesthesia in the companion canine model of osteosarcoma pain (Karai et al., 2004; Brown et al., 2005; Brown D. C. et al., 2015).

### Delineating the Site of Action of RTX

At the time of these earlier experiments, the intrathecal actions of RTX were attributed to deletion of TRPV1-expressing DRG neurons. However, further studies in rat, dog, and human revealed that when RTX is administered into the spinal CSF the most vulnerable neural structures are TRPV1-containing axons of Aδ- and C-fiber in the dorsal roots that are bathed in spinal CSF. In fact, the neuronal cell bodies and their axonal connection to the periphery are largely spared in comparison to the centrally projecting axons in the cauda equina. These issues are examined in detail in Sapio et al. (2018) and the mechanism of axonal susceptibility now governs the mechanics of intrathecal RTX administration (Sapio et al., 2018). These mechanistic insights point towards better results being obtained with small volumes of concentrated RTX, which in these studies can be optimized further by a slow infusion rate to keep the drug confined to the cauda equina and not distributed widely in the spinal CSF space (Shafer et al., 1998; Eisenach et al., 2003). The exact parameters continue to be evaluated in humans, but we anticipate that, by limiting dispersion of the drug solution, stronger analgesia will be obtained with less thermal denervation outside the area of interest.

### The Value of a Transitional Model: Lessons from Resiniferatoxin

We have mainly described the mechanistic and preclinical rodent studies that led to the initial work with RTX. However, this work was done in parallel with canine studies which gave invaluable insight into the utility and usage of the drug. In the next sections, we describe the contributions of these transitional studies. The majority were conducted in companion canines with naturally occurring disease, but we will also address the advances that came from pig and other non-rodent animal models.

### Development of the Canine Brief Pain Inventory and Other Scales

One of the key elements in preclinical animal studies is the selection of a valid and informative analgesic endpoints. In the canine, pain evaluation tools have been developed and validated to allow usage by the research community. It is important to note that many elements relevant to the impact of pain on “life experiences” are not captured by pain rating scales alone. This includes the ability of pain to interfere with the activities of daily living and estimates of overall improvement in the well-being and quality of life of the companion animal. In humans these elements are measured as part of the human Brief Pain Inventory (hBPI). To capture these elements in an evaluation methodology, a canine Brief Pain Inventory (cBPI) was developed and validated by Brown and others in several reports (Brown et al., 2008; Brown et al., 2013a; Giuffrida et al., 2017). Additional questionnaire instruments were also developed including the Canine Owner-Reported Quality of Life assessment (CORQ) (Giuffrida et al., 2018). Development of these instruments was critical to the methodological standardization of the companion canine model in pain research. The cBPI was validated to evaluate chronic pain in two disease states osteoarthritis Brown et al. (2008) and bone cancer Brown et al. (2009). These were followed by cBPI measurements of reduction in pain intensity and interference with activity in a series of cancer pain studies evaluating RTX and substance P-saporin Karai et al. (2004), Brown et al. (2005), Wiese et al. (2013), Brown D. C. et al. (2015), Sapio et al. (2018) and for evaluation of carprofen for osteoarthritis (Brown et al., 2008; Brown et al., 2010; Brown et al., 2013a; Brown et al., 2013b; Brown, 2014). The cBPI was ultimately used as the primary outcome for the pivotal studies that lead to the approval of Galliprant® (a small molecule EP4 antagonist) and Librela ® (an anti-NGF monoclonal antibody) for the treatment of pain in dogs with osteoarthritis. For analgesia studies, the cBPI scales were instrumental in quantitating the amount of pain relief in an open label study of RTX administered intraarticularly for canine osteoarthritis Iadarola et al. (2018) and by the intrathecal route in a double-blind clinical trial of bone cancer pain (Brown D. C. et al., 2015). The translational aspects of these two research efforts and problems encountered are treated in more detail in the next sections. It is important to recognize that in both cases RTX successfully transitioned to the IND and clinical trial stage and beyond. Nonetheless, many literature reviews of how to improve translational pain research often mention use of larger animal models but rarely include these examples. Canine companion models are still underutilized given their predictive utility and ability to enable clinical research. While preclinical rodent models are not replaceable, incorporation of a companion canine study can facilitate translational advancement in a more tangible way. The advantages of the companion canine model will be outlined in the following sections (see Table 1), as well as barriers that prevent the wider usage of these models. These barriers include the need for personnel to recruit and screen candidate patients, the requisite expertise and veterinary care facilities required to execute treatment and evaluation successfully, and the costs of canine studies. Nevertheless, a compelling case exists for how these studies can generate more predictive preclinical data that facilitates more rapid and reliable translation to new therapeutics (see Figure 1).

### Activity Monitoring as a surrogate endpoint for pain and evaluation of oral NSAID analgesia

Importantly, the subjective measures can be augmented with more objective methodology such as activity monitoring Dow et al. (2009), Michel and Brown (2011) and force plate measurement for weight bearing (Iadarola et al., 2018). For the endpoint of activity, use of a wearable monitor was evaluated in a randomized placebo-controlled analgesia study conducted in 70 arthritic dogs. Activity was acquired at one-minute intervals continuously for 21 days: 7 days prior to and 14 days during treatment with the NSAID carprofen (Brown et al., 2010). The activity monitor detected significantly more activity during the carprofen treatment phase than at baseline. The activity monitor was able detect improvements in activity with respect to baseline in the carprofen group and the difference in improvement between the placebo group and the carprofen group. There are several notable elements related to this study. First, the ability of the companion model to differentiate placebo from active drug indicates detection of a true analgesic action. Second the companion model was useful in detecting analgesia using an orally dosed drug, rather than the single administration RTX type of interventional agent. Thus, the model appears suitable for assessment of more conventional orally bioavailable drugs as well as novel treatments Lascelles et al. (2018) and two additional successful development programs were noted in the preceding section. The use of activity monitors or other wearable monitors is an evolving prospect for analgesia studies. Important factors to consider are the proper data to collect from activity monitors, which algorithms to use, how to interpret changes, whether to monitor during daytime or nighttime or during sleep, and with or without a companion dog, for example, are all important parameters for these technological innovations. They represent important techniques that can augment typical questionnaire-based evaluations of analgesics with objective endpoint whether applied to humans or animals.

### Treatment of Naturally Occurring Bone Cancer Pain by Intrathecal RTX in the Canine Companion Model

Osteosarcoma is a fairly common cancer in large breed dogs and generally localized to the appendicular bones (Morello et al., 2011). Thus, two routes were chosen: intracisternal for fore limb tumors and lumbar cistern for hind limb tumors (Brown D. C. et al., 2015; Sapio et al., 2018). While is it possible to thread a catheter from the cisterna magna to the desired level of the cord Marsala et al. (1995), preliminary studies showed that needle access to the intrathecal CSF space at both sites was straightforward and could be reliably performed percutaneously without touching the spinal cord itself. A series of three cancer pain studies were performed in companion canines (Karai et al., 2004; Brown et al., 2005; Brown D. C. et al., 2015; Sapio et al., 2018). The first in 2004 (see Figure 2), a larger follow up in 2005, and a randomized single-blind study in 2015 of 72 animals. Two additional studies examined target engagement, site of action and histopathology (Hockman et al., 2018; Sapio et al., 2018). In the two early studies Karai et al. (2004), Brown et al. (2005), significant analgesia was observed that was durable despite progression of the bone tumor with some dogs being evaluated out to 3.5 months. In addition, after RTX treatment many animals (12 out of 18, 66%) were able to have reduced or completely eliminated their concurrent analgesic medications which consisted of non-steroidal anti-inflammatory drugs, steroids and opioids. The target engagement studies showed overall consistent results which included 1) loss of molecular markers in spinal cord reflecting loss of TRPV1+ nerve terminals in dorsal horn (e.g., substance P, CGRP or TRPV1 staining), 2) analgesia to thermal stimuli or decrease in cancer pain, 3) minimal drug-related adverse events, and 4) minimal toxicity to non-TRPV1+ neuronal cell bodies in the DRG (Hockman et al., 2018; Sapio et al., 2018). There was some evidence for neuronal loss, which was anticipated, but it was less than expected, which is consistent with retention of the majority of TRPV1+ neurons in the DRG. These observations are the basis for identifying TRPV1-containing axons in the dorsal roots as the most vulnerable neural structure, and that selective chemoaxotomy in the CSF space is the basis for RTX analgesia (Sapio et al., 2018).

The double-blind study used a time-to-event analysis as the primary endpoint, evaluating how long it took for dogs to require additional standard-of-care intervention following IT injection of RTX or placebo. In this study certain procedural effects occurred, and a detailed examination of them provides insight into experimental complications that can be encountered with the companion canine model and how to anticipate and avoid unexpected events. Apparent spinal headache symptoms in some dogs, which did not improve until the second week post injection confounded the short term cBPI assessment. The apparent post-dural puncture headache was seen in 17 out of 18 animals treated by the intracisternal route and may have influenced owner evaluations of pain severity or interference related to osteosarcoma pain in the first 2 weeks. As a consequence, early treatment-related improvements were not significant. Despite this impediment to cBPI measurements, significant differences at later time points were detected for analgesic endpoints such as 1) improvement in lameness (rated by a veterinarian blind to the treatment rather than the owner) and 2) time to unblinding in order to obtain additional analgesic treatment (significantly shorter for the vehicle animals). The important point is to recognize that RTX is an interventional agent, and any procedural problems can affect drug performance and impact pain evaluations especially at early post-injection time points.

It was, therefore, suggested that trial designs with interventional agents should include extended periods of evaluation as part of the primary outcome and multiple independent endpoints for determination of analgesic actions to account for any variability due to administration parameters. Also, progression of the disease can be an issue if bone tumors or arthritis develops outside the zone of initial drug delivery. If the candidate analgesic is an oral drug, evaluation is less likely to be influenced by procedural parameters. However, other factors such as drug adsorption, metabolism and pharmacogenetics need to be incorporated and consideration given to behavioral and differences among breeds (Ostrander et al., 2017; Bowden et al., 2018). Caregiver placebo effects associated with an owner-evaluator are another factor that needs to be monitored. Lastly, the influence of expectations and pre-conceived bias with respect to breeds and pain sensitivity may need to be taken into consideration (Gruen et al., 2020). These details are enumerated to reinforce the idea that the interplay between trial design, owner evaluations, use of the canine BPI, procedure-related effects, analgesic activity, timing of endpoints, and use of independent trained observers all need deliberate consideration at the start of a proof-of-concept efficacy trial.

### Treatment of Human Cancer Pain by Intrathecal RTX

**FIGURE 3:**
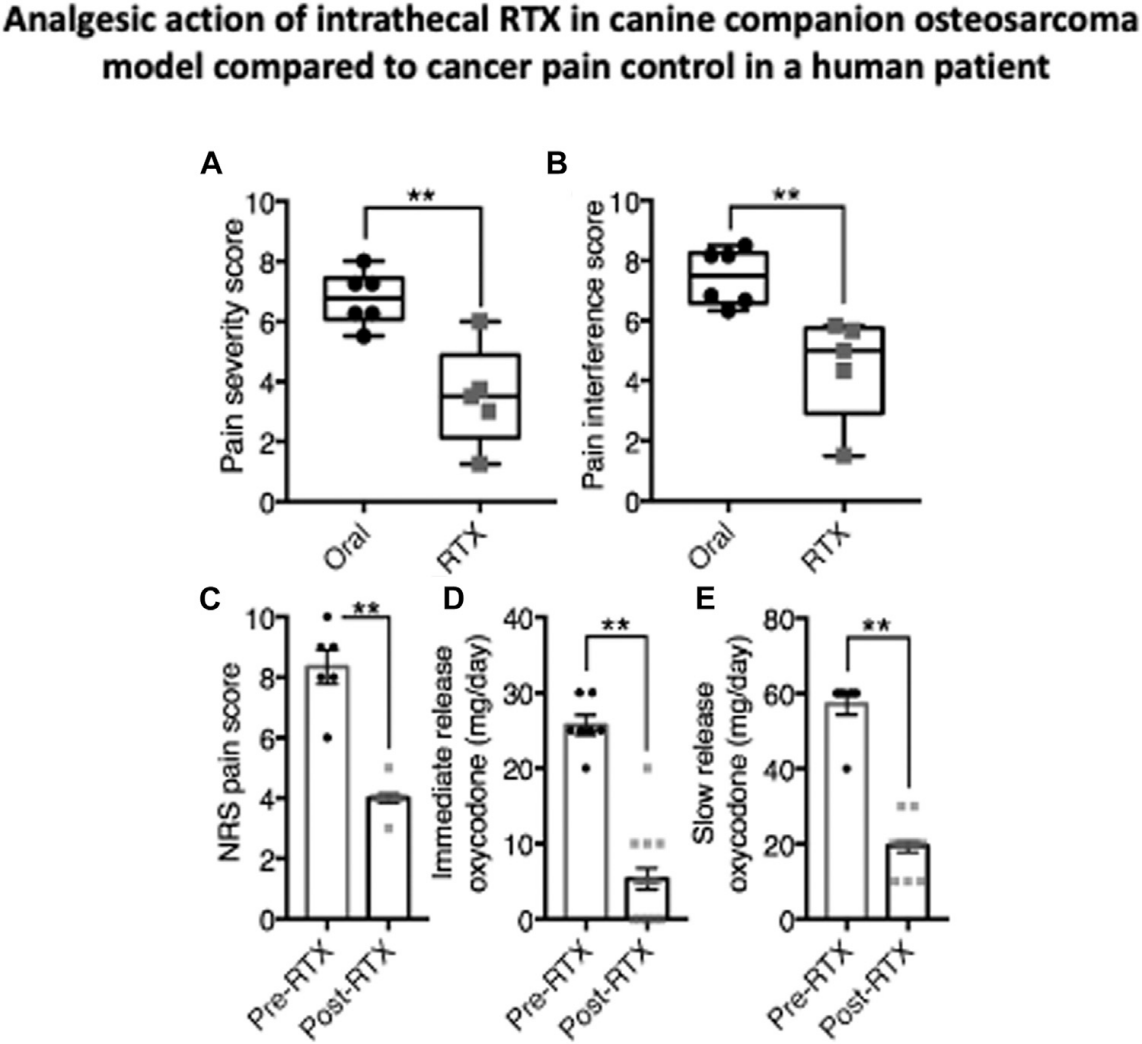
Analgesic action of intrathecal RTX in companion canine osteosarcoma model. compared to cancer pain control in a human patient. Dogs with appendicular osteosarcoma (*n* = 6), and a human patient with intractable cancer pain (*n* = 1) were injected intrathecally with RTX (1.2 μg/kg and 13 μg, respectively). Subsequent to IT RTX treatment, dogs were released to their owners and pain assessments were made at monthly intervals. Owners for each dog rated pain severity and interference with function using the Canine Brief Pain Inventory (79). Pain Severity **(A)** and Pain Interference **(B)** scores for companion dogs treated with RTX were significantly decreased relative to dogs kept on traditional standard of care oral pain medications alone. Data from the one human patient were collected over 7 days before RTX and 15 days after RTX injection while an in-patient on the oncology ward. Beginning immediately after RTX treatment, NRS pain scores (0–10) decreased from about eight to about 4 **(C)** Concurrently, self-administration of immediate release oxycodone was decreased **(D)** as was slow-release oxycodone during the same period **(E)** Statistical comparisons were made using two-tailed Mann-Whitney U test (**, *p* < 0.01) (data from Sapio et al., 2018, with permission). The analgesic effect in the human is remarkably similar to that seen in treated dogs in Figure 2 and reductions in canine analgesic drug treatments are also observed (Brown et al., 2005; Brown D. C. et al., 2015).

Two studies of RTX to treat intractable pain in advanced cancer have been conducted, one using an intrathecal route and the other an epidural route. Preliminary data on for the intrathecal trial are available in abstract form Heiss et al. (2015), Pomeraniec et al. (2020) and preliminary epidural results can be found at <https:// www.cancernetwork.com/view/interim-analysis-phase-ib-study-resiniferatoxin-generates-positive-data>. Additionally, molecular and analgesia data were published from one intrathecal human patient whose DRG and spinal cord were obtained after autopsy (Sapio et al., 2018). This patient’s data are presented along with data from a small cohort (N = 5) of companion canine dogs with osteosarcoma analyzed in parallel (Figure 3). The dogs exhibited significant decreases in their pain severity and pain interference scores post treatment. Similarly, the human exhibited a 52% decrease in pain severity and similarly pronounced reductions in opioid use. These results exemplify the strong analgesic response that is possible with intrathecal RTX in pain from advanced cancer.

## Protein Therapeutics for Cancer Pain

### Substance P-Saporin and Substance-P *Pseudomonas* Exotoxin

The companion canine model has also been used to evaluate another type of long-acting pain control agent; a protein therapeutic called substance P-saporin (SP-SAP) (Brown and Agnello, 2013). This compound, and a similar conjugate of Substance P with a truncated, bioengineered *Pseudomonas* exotoxin (SP-PE35) Iadarola et al. (2017), act on second order spinal cord neurons expressing the receptor for substance P, the Neurokinin one receptor (NK1) coded for by the TACR1 gene. Substance P is an 11 amino acid, C-terminally amidated peptide released from primary afferent endings onto NK1-receptor-positive spinal cord neurons. The agonist-bound receptor complex in internalized into the neuronal cytoplasm in clathrin-coated vesicles in a process referred to as agonist-mediated endocytosis (Mantyh et al., 1995). After internalization, the two protein toxins are released from the endosome into the cytoplasm and their enzymatic activity stops protein synthesis. Without protein synthesis the NK1+ neurons die, eventually removing these critical populations of neurons from the nociceptive transmission pathway to the brain. In preclinical rodent studies intrathecal injection of the two agents resulted in analgesia to a variety of experimental stimuli or hyperalgesic states (Mantyh et al., 1997; Wiley, 2008).

### Treatment of Naturally Occurring Bone Cancer Pain by Intrathecal Substance P-Saporin in the Canine Companion Model

Three studies have been conducted with this reagent in the dog. Two published reports evaluating the toxicology and basic neurobiology of SP-SAP in dogs provided positive information on dose response and target engagement, such as loss of NK1 receptor expressing neurons in the dorsal spinal cord. The third report examined pain control in companion canine dogs with pain from bone cancer using the intrathecal route of administration (Allen et al., 2006; Wiese et al., 2013). In two of the studies some animals exhibited a motor function deficit (e.g., motor weakness) which prompted euthanasia in several cases. The basis for this needs further study. This is mentioned to reiterate the point that these dog studies can be subject to experimental variables like any other interventional drug assessment. However, in the companion study, despite procedural variables that may have affected owner’s early assessment of pain using cBPI instruments, two other endpoints were acquired as the primary outcomes (Brown and Agnello, 2013). These measured the timing of, and the necessity for, unblinding and analgesic dosage adjustment. The two parameters were significantly different between the control group versus SP-SAP treated animals which is consistent with an analgesic action. In this experiment the SP-SAP was administered into the cisterna magna for fore limb tumors and at the lumbar enlargement of the spinal cord for hind limb tumors, thus the location of drug administration matched the location of the relevant spinal cord NK1+ neurons. SP-PE35 has not been tested in canines yet. These canine studies formed a major translational foundation for a human clinical trial.

### Treatment of Human Cancer Pain by Intrathecal Substance P-Saporin Conjugate

A small cohort of cancer pain patients (*n* = 3) was treated with SP-SAP and reported in two abstracts (Frankel et al., 2014; Frankel et al., 2016). This trial commenced at a no observable effect level dose and did not reach doses that had a clinical analgesic effect. The amounts tested were 1, 2, and 4 µg which were not in the range of the effective doses in the dog studies (∼20 µg and higher). In addition, the SP-SAP was delivered into the lumbar cistern. While this is the accepted route for a lumbar puncture it is somewhat remote from the location of the relevant NK1+ neurons in the superficial dorsal horn of the lumbo-sacral cord. In fact, the lumbar cistern is ∼30 ml s, and while some volume is taken up by the dorsal roots, this site likely caused a dilution of the SP-SAP which also may have contributed to a lack of analgesic action. It seems that with some adjustment of the dosing and delivery parameters to more closely match the canine studies, a resumption of the clinical trial would be worthwhile.

### Treating Osteoarthritis Pain

#### An Example of Translational Progression Using the Canine Companion Model:

**FIGURE 4:**
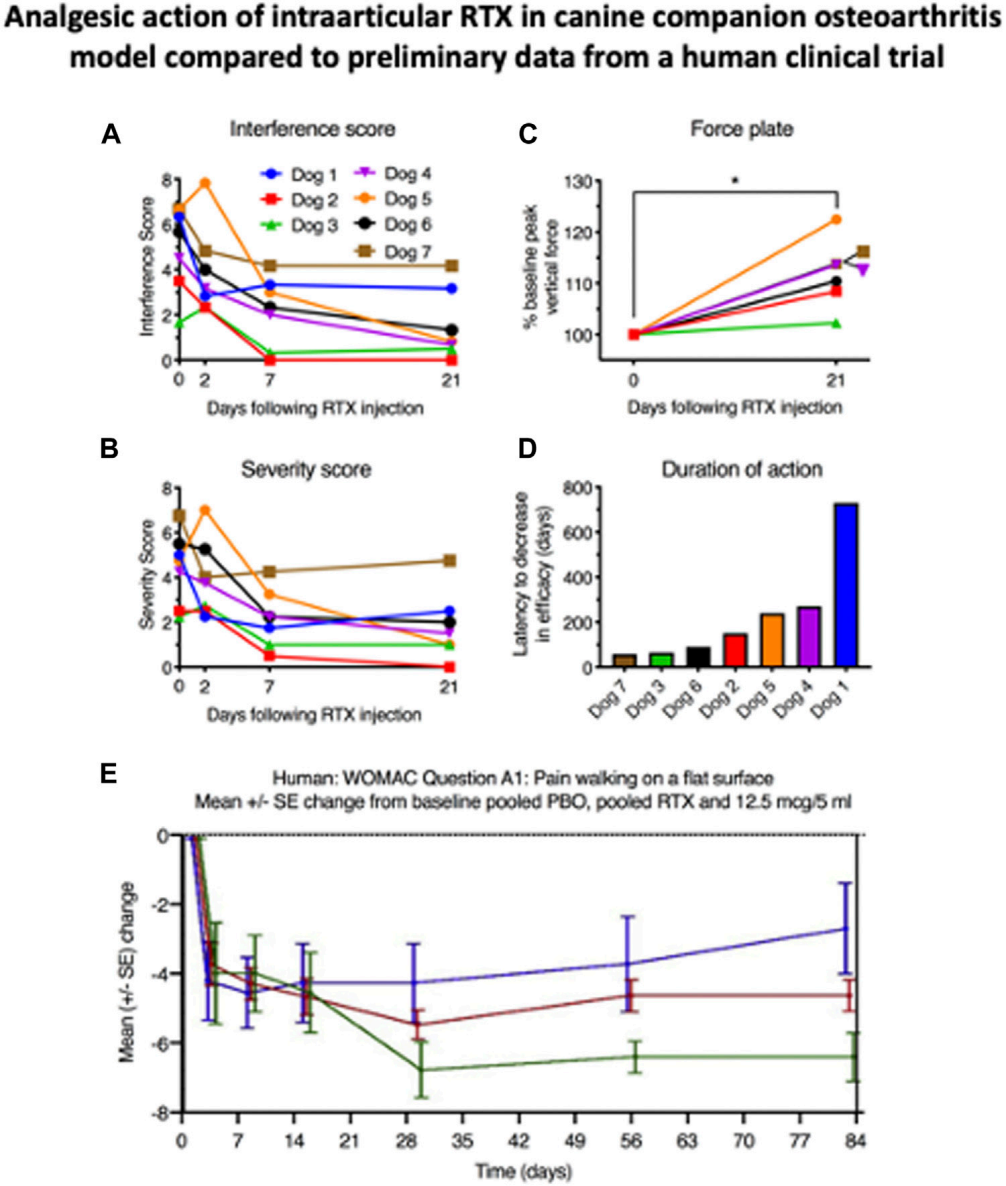
Analgesic action of intraarticular RTX in companion canine osteoarthritis model compared to preliminary data from a human clinical trial. These five panels show the fluid transition from the companion canine model to a human osteoarthritis clinical trial. In both species RTX was delivered intraarticularly (fore or hind limb in the dog and into the knee joint in the human). The canine work is from reference 37, the human clinical data are adapted from a published abstract (Leiman et al., 2020). Note the duration of the 5 ml placebo injection which only begins to wane after 14 days despite the fact that only one injection of either vehicle or RTX was delivered reducing the opportunities for reinforcement such as daily oral medication which are absent with this interventional single injection treatment.

The progression of research from rodent to canine to human is exemplified by the development of RTX for the treatment of osteoarthritis pain. It was evident from our initial RTX studies in 2004 that this compound was a very versatile interventional pain control agent suitable for multiple pain indications. In the rat, in addition to analgesic and anti-hyperalgesic actions of RTX on hind paw inflammation Neubert et al. (2003) or hind paw incision Raithel et al. (2017), intraarticular RTX had been shown to inhibit carrageenan-induced knee inflammation (Kissin et al., 2005). While these studies succeeded as “activity detectors” and gave an idea of a starting dose, they did not predict performance in pain from naturally occurring osteoarthritis in either canines or humans. Just prior to intraarticular RTX, an injection of bupivacaine was administered into the index joint to block the acute nociceptive actions of the 10 µg dose of RTX (Iadarola et al., 2018). Using the cBPI scales we detected decreases in pain severity and interference scores across all animals (Figures 4A, B). The onset could be seen by 2 days in most dogs and significant decreases were obtained at days 7 and 21. There was also an increase in weight bearing by the treated limb, an objective endpoint (Figure 4C). What was remarkable, was the long duration of therapeutic effect obtained from a single injection: suppression of pain, improvement in gait, weight bearing, and improvement in the dog’s activities of daily living all lasted 4 months or longer. The median time at which the owners sought re-treatment for their animal was 5 months and one dog displayed pain relief for more than 1 year (Figure 4D). Two to 3 years after injection there was no indication that the period of decreased TRPV1 innervation accelerated joint degeneration (Charcot joint) in any of the dogs. These strong positive results in naturally occurring OA were very incentivizing (see video: http://links.lww.com/PAIN/A627) (Iadarola et al., 2018). It must be pointed out that this was an open study and while not placebo controlled, the attached video, and other videos sent by the owners depicting robust spontaneous activity, mitigates attributing all of the improvement to placebo. However, in the human (Figure 4E), the placebo effect is both strong and of long duration, even though there was only one treatment session (discussed below).

The compelling efficacy of intraarticular RTX in the companion canine model, without apparent localized side effects, formed the impetus for toxicological studies in purpose bred dogs and prompted a full development program for control of clinical signs of osteoarthritis pain in companion animals, by one of the authors (AN, unpublished), in parallel to human clinical trials. In these studies, while the dogs can display short-term responses to drug administration (feeling hot, panting and salivating) no joint toxicity was observed. Furthermore, a group of aged beagles with light to moderate OA (not requiring NSAID treatment) was treated in the hip joint with no safety signal of concern. These observations in aged and laboratory dogs inform about the safety of the drug locally and form a path for further studies in humans beyond the knee joint. The lack of observable toxicity at the therapeutic dose for the animals or at the cellular level for the joint capsule provided essential information for submission of the investigational new drug (IND) application to the FDA. The question posed for the current topic is: How well did the companion canine results predict efficacy when translated to the human? In the case of RTX, the information gathered through companion animal studies provided much value and helped accelerate the human drug development program compared to traditional expected timeframes.

### Treatment of Human Osteoarthritis Pain by Intraarticular RTX:

After the IND milestone was reached, a double-blind placebo-controlled phase I trial was designed and conducted in humans (Leiman et al., 2020). The starting doses (between 3 and 30 µg delivered in five or 10 mls of saline) were justified from the dog studies, although the volume is considerably larger in this human study than in the initial companion work (Figure 4E). Efficacy was measured with the Western Ontario and McMaster Universities Arthritis Index (WOMAC scale) and a numerical pain rating scale. Commencing at about day 21 differentiation from the placebo injection began. At the 4-weeks evaluation point, the 12.5 µg dose was significantly different from placebo (green line, Figure 4E). The analgesia was sustained out to 12 weeks, and anecdotally, as long as 1 year. No serious adverse events occurred and the adverse events that were observed were minor, transient, and did not correspond to dose escalation. While these data are preliminary, the correspondence between the dog and human studies is apparent: Both showed strong efficacy and long duration. Obviously further follow-up is needed in both species but at least preliminarily, effective translational prediction from the companion canine model is substantiated.

One difference between the canine and human osteoarthritis studies was the long duration needed to differentiate the active drug arms from placebo, which was about 3 weeks in humans. This is a substantial difference from the dog study where onset of action was discernable in 2 days. This was an open label study and comparison to vehicle controls was not possible. There may be a myriad number of explanations for this difference. One factor is that the placebo response in human pain studies is known to produce a strong analgesic action regardless of the treatment. However, with tanezumab, an anti-NGF antibody, patients with osteoarthritis pain experience an analgesic response that differentiates from placebo as soon as 1 week after treatment. It is possible that other factors, perhaps related to intraarticular instillation of the vehicle, might have produced short term actions that are additive with the placebo response. Comparison to just a needle insertion into the joint was not made. More studies will help to resolve some of the administration procedural elements that shape the human response. The important point is that effective and long-term analgesia was obtained in the human and these two therapeutic characteristics were predicted using the translational companion canine model. Additionally, other important information is generated during the course of the canine trials. These include procedural steps such as anesthesia, justification of starting dose, type of interventional administration protocol, general side effect profile, and duration of action, which is a critical factor for determining dosing interval and how long to conduct the study are all robustly anticipated. Thus, the companion canine model provided actionable data on potential clinical efficacy in humans in two different clinical pain conditions, osteoarthritis and advanced cancer, and was a key element in guiding the expenditure of effort to reach the stage of human clinical trials.

### Swine Model for Interventional Analgesics

#### Fine Tuning Periganglionic or Intraganglionic Routes of Administration

Preclinical swine models are preferred in cases when the injection technique and/or anatomical precision is critical to the success of an experimental therapy. Recent advances have led to an increase in needle guidance and precision medicine technologies to deliver local analgesics and lesioning agents (Wood et al., 2007; McKnight et al., 2020). As the capability to deliver injections directly into the DRG grows, RTX stands out as an ideal candidate for injection in or around the ganglion. Several elements such as a strongly lateralized pain problem, dermatomal locations of a tumor or injury above the cauda equina, motivate the more localized approach of drug delivery directly to DRG neurons than afforded by intrathecal or epidural injection routes. Work towards optimizing periganglionic administration has been evaluated in a pig model. Close ganglionic injection of small volumes and amounts of RTX to lumbar DRGs on one side of the pig’s body selectively ablated TRPV1+ pain fibers unilaterally (Brown J. D. et al., 2015). Four ganglia were treated with vehicle on the other side. Control ganglia were unaffected neurochemically and behavioral responses to thermonociceptive stimuli were also intact on the vehicle side. This method is ideal for applications where the peripheral generator is driven by an identifiable ganglion or ganglia that can be individually targeted with precision guided injections and could also be appropriate for unilateral pain problems where ablating bilaterally would be unnecessary (e.g., postherpetic neuralgia). One pain indication for which direct ganglion targeting can be used is trigeminal neuralgia, an extremely painful disease the has origins in the trigeminal ganglion. While the exact etiology is not fully understood for trigeminal neuralgia, RTX may be an ideal candidate for an injectable therapeutic agent and may be more beneficial than other types of non-specific, ablative procedures such as radiofrequency lesion. For the patients and the interventionalist, selectively targeting TRPV1 neurons in the trigeminal may provide a permanent therapeutic response, which is important given the technical requirements of the injection (Tender et al., 2005). Here again the data from the large animal model supports the preliminary preclinical results obtained in rodent models and reinforces the confidence for obtaining a positive analgesic response in a human clinical trial.

This idea is bolstered by recent studies supporting the role of the peripheral generator in the etiology of several types of pain, including phantom limb pain, which was previously hypothesized to have a central origin. Recent studies have shown that lidocaine inhibition of the ganglia can prevent pain in the phantom limb, strongly supporting a DRG location for the peripheral generator in this pain condition (Vaso et al., 2014). This has been investigated with lidocaine or bupivacaine injections to “find” the source of the pain problem followed by ablation to extend the duration of the analgesia. These methods have seen success through ablation of an active neuroma (in cases where it could be identified), nerves, and nerve roots proximal to the ganglion depending on the patient (Ramanavarapu and Simopoulos, 2008; Wilkes et al., 2008; West and Wu, 2010; Imani et al., 2012; Zhang et al., 2017). As these patients are rare, most of this work has been done in case reports or in clinical practice, and more studies are needed to refine effective approaches based on evidence. However, RTX is ideally suited to replace radiofrequency or other ablative methods in this and other applications, as it spares normal sensations. These studies also reinforce the role of a peripheral generator in what was previously considered a form of central pain.

### Anecdotal “Very” Large Animal Experiences

#### Goat and Horse

**FIGURE 5:**
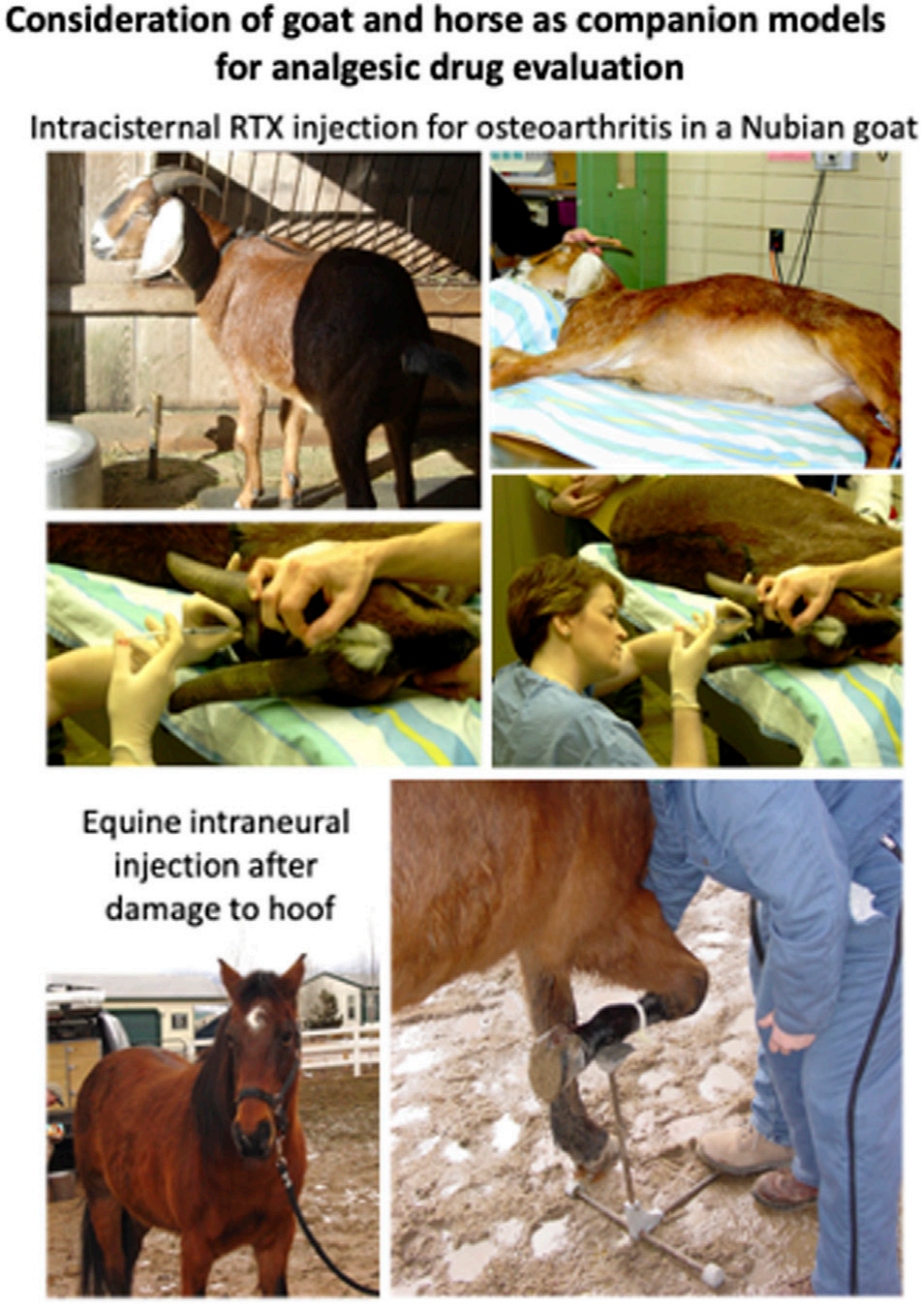
Consideration of goat and horse as models for analgesic drug evaluation. As discussed in the article, painful conditions in the horse and goat represent other opportunities for animal analgesic assessment. The goats were treated intrathecally for painful osteoarthritis by RTX injection into the cisterna magna by one of the authors (DCB). This produced noticeable improvements in activity. There were only two goats that had the arthritic problem, and this highlights the consideration of incidence and consistent access to a population with the appropriate disorder. The horse in the example was treated intraneurally with RTX and is shown just prior to surgical exposure of the nerve. This injection also produced a long duration analgesia (∼3 months) but it was not permanent (as expected with a distal peripheral injection). Compared to the goat, many more horses are candidates for such treatment, for example, laminitis is estimated at 10% of the equine population (Pollard et al., 2019). Other species have also been discussed in the literature (12, 13) and present challenges unique to each species.

Using models based on other, larger animals is another topic that is raised frequently in reviews of animal models for analgesic drug development. Table 2 lists a selection of studies that have used the companion canine model. At present a limited amount of experience treating pain with RTX in larger animals, which so far are horses and goats (Figure 5). These few cases, albeit anecdotal, are instructive. The horse in Figure 5 was a working horse and a source of livelihood for the owner. It became incapacitated by pain in the lower forelimb subsequent to an injury. RTX treatment was by intraneural injection. After establishing a dense local anesthetic block proximally, the nerve was surgically exposed and RTX was delivered intraneurally. There were no adverse events and the horse returned to work and could be mounted and ridden. Unfortunately, pain behaviors returned after about 3 months and the animal was euthanized. The goats that were treated came from a herd of Nubian goats located at the Bronx and Queens Zoos in New York. They developed osteoarthritis subsequent to infection with a caprine retrovirus (Crawford et al., 1980; Crawford and Adams, 1981; Blacklaws, 2012; Gomez-Lucia et al., 2018). The animal illustrated had arthritis in the forelimbs and was injected with RTX intracisternally while under isoflurane anesthesia. Recovery was uneventful and the goat returned to his exhibit space. Reports from his keepers indicated that considerable function had been restored: “Yesterday Simon (the goat) was head-butting other goats and people … something he hasn’t done for some time. It is the impression of the staff that he is doing fantastically. They are really pleased.” “The latest update: Simon has been seen doing things that he has not done for some time–standing with his front legs up on the fence; being up on a stump…. All in all, the Zoo staff is VERY impressed with his response.” A second arthritic goat was treated, with similar therapeutic response. These were preliminary pilot studies and the results, while demonstrating robust analgesia, remain unpublished but could be extended with a controlled study in settings where more animals need treatment.

**TABLE 2:** Examples of canine companion and other large animal models in pain research 2004-present.

Publication	Species, pain indication and technical aspects	Comments
Karai et al. (2004)	Experimental models in rats after intra-trigeminal or intrathecal RTX and in dogs with osteosarcoma, drug formulation, dose-ranging	First *in vivo* demonstration of intrathecal RTX analgesia in canine companion models of osteoarthritis and osteosarcoma pain
Brown et al. (2005)	Companion dog, unilateral osteosarcoma, dose response, hemodynamics and analgesia	Efficacy demonstrated for intrathecal RTX in cancer pain, reduction in use of other opioid and non-opioid analgesics
Tender et al. (2005)	Rhesus monkey, intra-trigeminal RTX as a model for control of facial pain, head and neck cancer pain, and trigeminal neuralgia	Unilateral microinjection into TG. Target engagement shown by ipsilateral loss of capsaicin induced eye wipe and plasma extravasation. No Adverse effects on skin, wound healing, feeding
Brown et al. (2007), Brown et al. (2008), Brown et al. (2009), Brown et al. (2010), Brown et al. (2013a), Brown et al. (2013b), Brown D. C. et al., (2015), Brown J. D. et al., (2015), Wiese et al. (2013), multiple additional publications	Development and validation of multiple metrics for canine pain in osteosarcoma and osteoarthritis	The brief pain inventory and other metrics were adapted and validated to evaluate spontaneous pain states in the canine companion model, objective endpoints such as activity monitoring, and evaluation of analgesic agents
Allen et al. (2006), Yaksh et al. (2017)	Canine toxicology studies of substance P-Saporin conjugate demonstrating target engagement and evaluation of adverse events	Examination of a ligand-directed plant protein that blocks protein synthesis in substance P receptor (NK1) expressing second order spinal cord neurons
Brown and Angello (2013)	Canine companion model, osteosarcoma pain, evaluation of substance P-Saporin delivered intrathecally	Veterinary clinical trial of a ligand-directed bioengineered pseudomonas exotoxin that blocks protein synthesis in NK1 expressing second order spinal cord neurons
Brown D. C. et al., (2015)	Double blind evaluation of RTX in canine bone cancer	A veterinary clinical trial of intracisternal or intrathecal RTX, owner and veterinarian evaluation of spontaneous pain and interference with daily activity
Brown J. D. et al., (2015)	Swine, experimental heat pain, unilateral lumbar peri-ganglionic infusion of RTX	CT image-guided delivery of RTX demonstrates lateralized confinement of injection], analgesia to laser heat pulse and loss of TRPV1 from ganglia
Iadarola et al. (2018)	Canine companion model, osteoarthritis pain, evaluation of RTX delivered intraarticularly	A single injection of RTX produced long-duration analgesia, increased activity with no side effects and without negative side effects
Maus et al. (2019)	Swine, technical evaluation of MRI for peri- or intra-ganglionic infusion of gadolinium contrast agent	MRI and contrast agent for visualization of needle placement and small infusion volumes into DRG. Potential for gene delivery
Guedes et al. (2017)	Horse, laminitis, evaluation of soluble epoxide hydrolase inhibitors as analgesics	Initial studies of efficacy for this prototype enzyme inhibitor

The horse has also been used for evaluation of other analgesics. An example being developed by Hammock and others are soluble epoxide hydrolase (sEH) inhibitors (Inceoglu et al., 2006; Wagner et al., 2017; Wagner et al., 2020). This approach centers around inhibition of the degradation of a class of arachidonic acid-derived epoxy fatty acids. The accumulation of these fatty acids is associated with better outcomes in terms of inflammation, primary disease progression, and pain. In addition to rodent models, this class of drugs has been evaluated in transitional models similar to RTX. One such study was done in companion canine animals with naturally occurring arthritis, which was evaluated primarily using the cBPI. This revealed sEH inhibition was effective in blocking arthritis pain in dogs (Wagner et al., 2017). Large animal studies of sEH inhibitors were initiated for treatment of severe refractory pain in horses with equine laminitis, a condition characterized by lameness requiring humane euthanasia (Guedes et al., 2013). The preliminary study was extended to a larger clinical case series, with both showing positive results and reinforcing the utility of the equine approach (Guedes et al., 2017)

### Molecular Transcriptome of Canine DRG

#### Molecularly-Validated Target Selection

We have described in this review that several models, particularly the dog and the pig have distinct advantages over the rodent in predicting efficacy, dosing and usage in humans. It is important to note the different ways the process of using the companion canine model builds on itself. In the canine intraarticular RTX osteoarthritis Iadarola et al. (2018) and intrathecal RTX bone cancer pain investigations Sapio et al. (2018), the molecular transcriptomics of the canine DRG were examined. These were the first studies to define the molecular biology of the DRG neuronal transmission step in the canine pain pathway. In the RTX osteoarthritis report, the complete molecular gene expression profile of canine dorsal root ganglion was determined by next generation RNA-Sequencing (Iadarola et al., 2018). These state-of-the-art results bring the molecular knowledge base for canine pain studies on a par with the human, rat and mouse. These studies allow for a complete molecular framework to be obtained and the “druggable nociceptome,” which contains all the genes that might code for proteins that transmit or modulate nociceptive transmission, to be identified. This gene can be bioinformatically extracted from the total transcriptomic library and used to design future analgesic drug trials. If contemplating a drug trial, especially a drug designed to act on primary afferent nociceptors, knowing whether the target molecule is expressed in the DRG of the animal model, as well as the human, is an extremely important consideration for selection of the model animal, and ultimately to the success of the trial. For example, clinical trials and animal studies of Kappa opioid receptor agonists may exhibit analgesic and anti-pruritic actions, particularly for visceral modalities (Kaski et al., 2021). However, while published studies have shown anti-pruritic actions of kappa agonism Fishbane et al. (2020), reports of analgesic effects remain largely unpublished as of 2021. More complicated peripheral agonists at delta-kappa opioid heterodimeric receptors have also exhibited analgesia in the rat. However, deep RNA sequencing of *canine* DRG shows that kappa opioid receptors are not expressed in canine DRG, a finding which would eliminate evaluation of peripheral kappa agents in a canine model. Thus, knowing the transcriptomic profile of dog DRG may help guide decision making and whether or not to incorporate the companion canine model into drug development effort.

## Further Adoption of the Canine Companion Model

It is disconcerting that, while bemoaning the lack of translational success using rodent models of nociception, neuropathic pain, cancer pain, and affective components of pain, the research community has been slow to adopt alternatives to the traditional approach. There is a recognized need for new animal models such as the companion canine model but, apparently, there is little interest in developing programs that utilize these types of approaches. This may be due in part to difficulties in establishing the research infrastructure, and in part due to the limited applications to which this type of animal research is relevant. For example, the mainstay mechanistic research performed in most university settings is generally not appropriate in larger animals due to costs, logistics, and ethics. The appeal of rodent models, and the fallacy of rodent models, lies in the perception of standardization and repeatability. However, it is debatable whether analgesic efficacy in a given homogenous rodent model is broadly predictive of the human translational value, due to both the heterogeneity of naturally occurring disease, the difference in metabolism and kinetics, and various other factors associated with individual humans and complexities of underlying degenerative disease processes. The canine transitional model provides accelerated assurance that the analgesic efficacy is translational. It can also be accurate if conducted following good clinical practices with control groups. Research on this level is very practical for developing evidence for *transition* from laboratory work into human clinical trials. Rather than discover new mechanisms, analgesia research in larger animals builds on the laboratory based mechanistic insight to ask an important question: “Is the compound an effective analgesic?” Even if using a novel reagent, results from such analgesia studies are frequently judged as deficient since they are perceived as not providing new mechanistic insight. This “insufficient novelty” not offset by the gain in new knowledge that the analgesic agent under consideration actually works. Unfortunately, the “mechanism” viewpoint may extend to grant reviews, as this kind of work may not be prioritized because of the prejudice against the value of a practical line of inquiry. Nevertheless, as we show in this review, the combination of positive results in rodents, with a positive outcome in a companion canine model constitute invaluable translational milestones for a successful outcome in human trials.

## Summary

### Translational advantages of the companion canine model

As noted, one of the subjects mentioned frequently as a possible remedy for the lack of translational success of mouse- or rat-based models was the inclusion of large animals into the repertoire of pain research. As we show in this review, evaluating drugs in larger non-rodent species can serve as an excellent “accretive second level of assessment” for efficacy and side effects and provides a strong set of observation for informed decision making about further drug development needs. One advantageous characteristic is that the pain-causing problems frequently arise from naturally occurring diseases or disorders and thereby are not really models at all, but bona fide pain problems that are very similar to the human disorders. We used dog osteosarcoma pain as part of the preclinical basis for our human phase I trial of RTX in cancer pain and for many other disorders. Several aspects that can influence the success of translational pain research are summarized in Table 3.

**TABLE 3:** Elements related to translational success.

**Transcriptional Validation** Is the target gene expressed in target tissue? In some cases, the foundational data needs to be verified. Solution: Measurement of gene expression levels for target molecule by RNA-Seq
**Neuronal and neural circuit cross-species validation** Are the neuronal populations in the model species the same as in human? Is the relevant neuronal population in PNS or CNS? Centrally, is the circuit “wiring” the same? Are the transmitters and peptides in the neurons the same? With peripheral tissue damage are the plastic changes in spinal cord the same?
**Peripheral validity** Are inflammatory mediators similar? Do they perform the same function? Are the local tissue transcriptomic changes the same? Are the infiltrating leukocyte populations the same? Is the timing of inflammation/damage-response similar? Again, some of the outstanding gaps can be filled by conducting RNA-Seq on the model (26,38), anatomical techniques. And methods that combine both such as spatial transcriptomics
**Types of preclinical models** Standard rodent inflammation and nerve injury models of various types + more specialized models such as burn, osteoarthritis and osteosarcoma. This is a vast literature. With judicious interpretation they are good initial “activity detectors.” As soon as larger animals become subjects the number of studies falls off precipitously. In the dog the primary models are osteoarthritis and osteosarcoma
**Interpretation of data from models** Dogs like other animals can be influenced by the testing environment (clinic or outdoors), who is in the vicinity or handling them and the presence of other dogs to interact with. These factor need to be accounted for in the study design and data interpretation and can be added into the testing protocol. Ideally, efficacy should be obvious. If complex statistics are needed to discern an analgesic effect, a similar situation will likely pertain to a human trial.
**Measurement Tools** Pain rating methods include the canine brief pain inventory and other scales and subjective evaluations. For pain disorders affecting the limbs locomotion and force plate measurements can be obtained. However, such testing can be painful if the pain from the underlying condition is severe, and this may make obtaining reliable pre-analgesic testing difficult to acquire and assess for comparison. Similar considerations apply to gait analysis
**Problems that May Be encountered** Damage to the injection site from the physical injection procedure can occur if the active pharmaceutical is administered by an interventional procedure. Orally administered drugs may cause aversive effects such as nausea that may not be obvious but influence testing. The main requirement is that the treatment produce a robust analgesia. Marginal analgesics may not produce a signal but theoretically could be active to a limited extent in humans

The questions that are pertinent to the companion canine model are: 1) What advantages accrue to the translation to human and/or veterinary patients? 2) How well will the information obtained translate to phase I trials and beyond? 3) How complex of a study design is needed and for how long? and 4) Will the study follow Good Clinical Practices guidelines? Clearly one advantage is pharmacological. The results obtained provide strong data for 1) dose justification 2) duration of action, 3) strength/efficacy of analgesia, 4) potential side effects, 5) potential for evaluation of drug interactions, and 6) other types of behaviors that may emerge while the dogs are in the care of their owners. The companion canine model has additional advantage of continual observation of the treated patient by one and often many observers. Both pain rating, daily interference, owner evaluation of behavioral metadata, and evaluation of spontaneous behaviors, can all be obtained remotely, and more specialize evaluations can be conducted at scheduled visits to the veterinary clinic. Assuming all goes well, the data obtained should be additive with preceding rodent data.

Lastly, evaluating a potential analgesic in the companion canine model has the potential to be a cost saving step in the development life cycle for industry. One example where the companion canine predicted failure in human clinical trials was that of TRPV1 antagonists, which were unsuccessful in the dog, and subsequently failed in clinical trials (Lascelles et al., 2018). This lack of efficacy in the canine model was an early warning sign that these drugs lack efficacy, as the molecular target is conserved in the dog, where it has a very similar function (Iadarola et al., 2018). An example of where the companion canine model could have been informative might be EMA401. This is an angiotensin II receptor two antagonist that showed some analgesic activity in a small double-blind placebo controlled clinical trial in human neuropathic pain. The clinical trial results did differentiate between placebo and active agent, although the difference was not that large. EMA401 was subsequently acquired by Novartis and, during more extensive trials, it failed to show analgesic activity. While it is hard to model human neuropathic pain in animals, if for example, no activity was seen in a canine companion cancer or osteoarthritis trial, it may have modulated the ∼400 million dollar investment Novartis made upon acquisition and testing of this early-stage agent. This is aside from transcriptome analyses which show a very low expression level of the *AGT2R2* gene in human or canine dorsal root ganglia. Another example is UBX0101 an anti-senescence drug. This molecule is an inhibitor of a protein-protein interaction between MDM2 and p53 where MDM2, which codes for a nuclear-localized E3 ubiquitin ligase acts as a negative regulator of the P53 tumor suppressor protein. Two human clinical trials of intraarticular injection of UBX0101 for osteoarthritis pain were conducted. The results were less than satisfactory. Aside from considerations of dose, pharmacokinetics, and schedule of administration as potential problem points, and assuming that the MDM2-P53 molecular interaction is similar enough in dog and human to support UBX0101 binding, a hypothetical case can be made for interposing a companion canine osteoarthritis study prior to the second phase two study (or earlier) as suggested in Figure 1. In this case, if the drug had failed to produce a robust analgesic effect or enhancement of daily living activities in canine osteoarthritis, then the project could have been terminated early and saved Unity Biotechnology and its investors approximately 80 million dollars. While these are hypothetical examples, the use of this model, especially for interventional approaches, can provide valuable performance information to make informed go-no-go decisions during a drug development program. This is not to say that problems are not encountered when using the companion canine model itself, as pointed out in both the RTX and SP-saporin sections and the potential for problems has to be recognized and guarded against as in any experiment. Another factor is having a proper alignment between the animal pain conditions and the intended human pain indication. While this may not always be possible, the many aspects of knowledge gained from a canine trial are generally applicable to evaluation of analgesic performance and potential side effect profiles across multiple pain indications.

In conclusion, interposing a large animal transitional model such as the companion canine model between the early stages of analgesic drug identification and the later stage of human analgesic clinical trial can provide many advantages. Obtaining an unbiased, objective assessment of analgesic activity, or lack thereof, is valuable, actionable information and a basis for guiding further development.
